# Supplementary material for: Performance evaluation of survival regression models in analysing Swedish dental implant complication data with frailty
Source: PLoS One. 2021 Jan 7;16(1):e0245111. doi: 10.1371/journal.pone.0245111 (PMC7790411; doi:10.1371/journal.pone.0245111)
Supplement: Attachment — Submitted filename: Response to reviewers comment MP KK JD.docx [file pone.0245111.s001.docx]

November 28th 2020

Dear Editor

PLOS ONE

Through

Feng Chen
Academic Editor

PONE-D-20-32590
Performance Evaluation of survival regression models in analysing Swedish dental implant complication data with frailty

We the authors of above mentioned paper appreciate the efforts and comments of the editor and the eminent reviewers. We have addressed all these comments. A point-by-point response to the issues in our revised manuscript is listed below.

Please note that we used the file with the tracked changes to describe where the changes were made.

Editorial Comments

When submitting your revision, we need you to address these additional requirements.

 1. Please ensure that your manuscript meets PLOS ONE's style requirements, including those for file naming. The PLOS ONE style templates can be found at

https://journals.plos.org/plosone/s/file?id=wjVg/PLOSOne_formatting_sample_main_body.pdf and

https://journals.plos.org/plosone/s/file?id=ba62/PLOSOne_formatting_sample_title_authors_affiliations.pdf

 Thank you. We have reformatted the manuscript in line with Plos one requirements

2. Please correct your reference of  "p=0.000" to "p<0.001" or as similarly appropriate, as p values cannot equal zero.

 Thank you we have changed all its appearance

3. We note that you have indicated that data from this study are available upon request. PLOS only allows data to be available upon request if there are legal or ethical restrictions on sharing data publicly. For information on unacceptable data access restrictions, please see http://journals.plos.org/plosone/s/data-availability#loc-unacceptable-data-access-restrictions.

In your revised cover letter, please address the following prompts:

a) If there are ethical or legal restrictions on sharing a de-identified data set, please explain them in detail (e.g., data contain potentially identifying or sensitive patient information) and who has imposed them (e.g., an ethics committee). Please also provide contact information for a data access committee, ethics committee, or other institutional body to which data requests may be sent.

This study is human research based on sensitive data which cannot be publicly shared. Data contain potentially identifying and sensitive patient information. The interested party is referred to the Swedish Ethical Review Authority (Etikprövningsmyndigheten), Box 2110
750 02 Uppsala, Sweden, [registrator@etikprovning.se](mailto:registrator@etikprovning.se), +46 (0)10-475 08 00 or Head of Institute, Peter Lingström, Professor of Cariology, Institute of Odontology, University of Gothenburg, <https://www.gu.se/en/odontology/about-us/contact>, [info@odontologi.gu.se](mailto:info@odontologi.gu.se), [peter.lingstrom@odontologi.gu.se](mailto:peter.lingstrom@odontologi.gu.se), +46 (0)31-786 2932.

b) If there are no restrictions, please upload the minimal anonymized data set necessary to replicate your study findings as either Supporting Information files or to a stable, public repository and provide us with the relevant URLs, DOIs, or accession numbers. Please see http://www.bmj.com/content/340/bmj.c181.long for guidelines on how to de-identify and prepare clinical data for publication. For a list of acceptable repositories, please see http://journals.plos.org/plosone/s/data-availability#loc-recommended-repositories.

 We will update your Data Availability statement on your behalf to reflect the information you provide.

Kindly see the comment above.

 4. Your ethics statement should only appear in the Methods section of your manuscript. If your ethics statement is written in any section besides the Methods, please move it to the Methods section and delete it from any other section. Please ensure that your ethics statement is included in your manuscript, as the ethics statement entered into the online submission form will not be published alongside your manuscript.

 Thank you. It has been moved

5. We note you have included a table to which you do not refer in the text of your manuscript. Please ensure that you refer to Table 3 in your text; if accepted, production will need this reference to link the reader to the Table.

 Thank you. It has been moved

Reviewers' comments:

Reviewer's Responses to Questions

**Comments to the Author**

1. Is the manuscript technically sound, and do the data support the conclusions?

The manuscript must describe a technically sound piece of scientific research with data that supports the conclusions. Experiments must have been conducted rigorously, with appropriate controls, replication, and sample sizes. The conclusions must be drawn appropriately based on the data presented.

Reviewer #1: Yes

Reviewer #2: Yes

Reviewer #3: Yes

Thank you all.

2. Has the statistical analysis been performed appropriately and rigorously?

Reviewer #1: Yes

Reviewer #2: Yes

Reviewer #3: Yes

Thank you all.

3. Have the authors made all data underlying the findings in their manuscript fully available?

The [PLOS Data policy](http://www.plosone.org/static/policies.action#sharing) requires authors to make all data underlying the findings described in their manuscript fully available without restriction, with rare exception (please refer to the Data Availability Statement in the manuscript PDF file). The data should be provided as part of the manuscript or its supporting information, or deposited to a public repository. For example, in addition to summary statistics, the data points behind means, medians and variance measures should be available. If there are restrictions on publicly sharing data—e.g. participant privacy or use of data from a third party—those must be specified.

Reviewer #1: Yes

Reviewer #2: Yes

Reviewer #3: No

Thank you all.

4. Is the manuscript presented in an intelligible fashion and written in standard English?

PLOS ONE does not copyedit accepted manuscripts, so the language in submitted articles must be clear, correct, and unambiguous. Any typographical or grammatical errors should be corrected at revision, so please note any specific errors here.

Reviewer #1: Yes

Reviewer #2: Yes

Reviewer #3: Yes

Thank you all.

5. Review Comments to the Author

Please use the space provided to explain your answers to the questions above. You may also include additional comments for the author, including concerns about dual publication, research ethics, or publication ethics. (Please upload your review as an attachment if it exceeds 20,000 characters)

Reviewer #1: This paper evaluated the performance of 13 survival regression models in analyzing dental implant complication based on a case study in Sweden. The topic is interesting and worth of investigation. The results indicate that the flexible parametric survival model with frailty outperforms the other alternatives. The findings provide a good suggestion on method selection for modeling dental implant complication. Overall, the paper is generally well organized and qualified to be published in PLOS ONE.

Thank you all.

Reviewer #2: This paper evaluated the performance of 13 survival regression models in assessing the factors associated with the timing of complications in implant-supported dental restorations in a Swedish cohort. The topic is interesting. The methods sound. The results are meaningful and useful. One only issue is that the resolution of some figures could be increased.

Reviewer #3: This paper is written in a way that takes the reader through the models very well.
Thank you all.
I have some minor comments:
I am not familiar with dental data, so I didn't have a good idea about what an event was, and what a complication was in the sentence of: line 223: "There were a total of 1,038 events during the observation period with 469 complications in single-record/single-failure data".

The complications considered in the present analysis included (i) loss of an implant, (ii) development of peri-implantitis and (iii) technical problems (lines 220-222). The occurrence of any of these were considered as an “event”. We added a sentence to the relevant paragraph (lines 223-224) for clarification.

line 225-230: Is the model improvement assessed in line with how complicated the model is? As in, typically in multivariate models a new term is only included if the improvement in model fit is significant, as a particular level of significance. Was this considered here? It may be worth stating that the degree of improvement is not relevant in selecting a best fitting model. Reading the results section it does seem like there was a consideration for how complicated the model was.

Thank you. We didn’t set for model complication. We set out to select the best model irrespective of its simplicity or complexity in as much it considered frailty.

line 285: is it relevant to have the p-value to 4 decimal places? Wouldn't 2 do?

Thank you, three decimal places are usually recommended by most journals including Plos One.

Table 3: are the estimates too precise? Would 2 decimal places for the hazard ratio and confidence intervals still provide the same relevant information, and p-values to 2 significant figures? (or 1 if p<0.01)

Thank you, yes, they will provide same information. Again, the journals requirement is 3 decimal places.
Also, present p=0.000 as p<0.001.

 Thank you we have changed all its appearance

6. PLOS authors have the option to publish the peer review history of their article ([what does this mean?](https://journals.plos.org/plosone/s/editorial-and-peer-review-process#loc-peer-review-history)). If published, this will include your full peer review and any attached files.


If you choose “no”, your identity will remain anonymous but your review may still be made public.


**Do you want your identity to be public for this peer review?** For information about this choice, including consent withdrawal, please see our [Privacy Policy](https://www.plos.org/privacy-policy).

Reviewer #1: No

Reviewer #2: No

Reviewer #3: No

Thank you all.

Adeniyi Fagbamigbe

On behalf of authors
